# Jottings

**Published:** 1910-02-12

**Authors:** 


### Jottings.

The Royal Free Hospital has received a gift of pheasants and also of cast linen from his Majesty the King, the Patron of the Hospital.

The Clackmannan County Secondary Education Committee has resolved that the medical inspection of school-children in the county be undertaken by only one fully qualified practitioner, with a salary of £325 per annum, inclusive of expenses.

The late Lady Campbell Clarke bequeathed a fund to the trustees and executors of her will, at their discretionary disposal, for charitable purposes; and out of which they have allotted a gift of £100 to the Newsvendors' Benevolent and Provident Institution.

Owing to the recent heavy snowstorms in the neighbourhood of Chicago the city suffered from a milk famine. Hospitals, children's homes, and asylums in several instances were without their daily supply, and physicians reported the famine to have had bad effect in patients needing the nourishment.

A General Court of the Governors of the Sunderland Infirmary was held at the Infirmary, on Wednesday, February 2, 1910, at eight o'clock p.m., to receive the reports of the General Committee and the auditor's financial statements; to elect officers, and to transact such other business as may be brought before the court.

The annual report of the National Dental Hospital states that last year Lord Portman headed the list of donations with a gift of £200. The committee is anxious to extend the accommodation and to provide adequate *x*-ray and photographic facilities, but these requirements would add another floor to the building, and the cost of this rearrangement would be about £2,000.

The Bury St. Edmund's and West Suffolk Sanatorium has now been opened for consumptives. The scheme was a practical idea for employing the money left in hand after the Bury Pageant. The building is of corrugated iron, lined with sheet felt at the back. There are two large wards—one for men, the other for women—each accommodating four patients.

The Rev. John Vincer Minter, the only workhouse and infirmary master known who is also a clerk in holy orders, has notified his intention of resigning that office, which he has held for 21 years, to take up the chaplaincy of the Barnet Union Workhouse and Infirmary. During his period of service at Newmarket he has shown the King and the late Duke of Cambridge round the institution.

The 121st annual report of the City Dispensary, Dowgate Hill, states that steady use is being made of this institution by the class for whom it is maintained, and 20,032 attendances on patients were made during the year. There were 47 surgical operations under anæsthesia and 13 urgent cases were attended to without letters of recommendation. The total income has been £912 13s. 3d., and the expenditure £932 18s. 2d.

Sir Savile Crossley presided at a special meeting of the board of the Hospital Saturday Fund Association, held last Saturday at the central offices, Gray's Inn Road, when it was resolved, on the recommendation of the distribution committee, that the sum of £28,671 3s. 10d. be awarded to the 214 participating institutions—namely, 34 general hospitals, £8,637; 15 cottage hospitals, £421; 76 special hospitals, £10,365; 30 dispensaries, £831; 24 convalescent homes, £1,534; 22 nursing institutions, £473; and 13 miscellaneous (including ambulance, distribution, surgical appliance, and executive committees), £6,410 3s. 10d.

The King has sent gifts of game to the Hospital for Sick Children, Great Ormond Street, to the Royal London Ophthalmic Hospital (Moorfields Eye Hospital), to the Royal Westminster Ophthalmic Hospital, to the Samaritan Free Hospital, to the Chelsea Hospital for Women, to the Royal Hospital for Incurables, Putney Heath, the British Home and Hospital for Incurables, Streatham, the Royal Ear Hospital, the East London Hospital for Children, Shadwell, and the Hospital for Epilepsy and Paralysis.

A special general meeting of the Governors of the Poplar Hospital for Accidents, Poplar, E., was held at the Hospital on Tuesday, February 1, 1910, at 4 o'clock, for the purpose: (1) Of appointing new trustees of the hospital; (2) of making such alterations in the rules of the hospital as shall be rendered necessary in consequence of such appointment; (3) of making such alterations in Rule 26 as may be thought advisable with regard to the investment of funds, and of defining the duties of the trustees in relation thereto.

The report for the year ended September 30, 1909, of the general committee of Addenbrooke's Hospital, Cambridge, states that the income, for the first time in the history of the hospital, rose to £10,000, and there is a credit balance of £287 on the year. There are, however, pressing needs for new wards for children and alterations to the administrative block, costing about £10,000, plus about £8,000, for maintenance, and of new laundry machinery at a cost of about £2,000. A new *x*-ray apparatus is being fitted up for £250.

